# Persistently Elevated Level of IL-8 in *Chlamydia* *trachomatis* Infected HeLa 229 Cells is Dependent on Intracellular Available Iron

**DOI:** 10.1155/2009/417658

**Published:** 2009-05-26

**Authors:** Harsh Vardhan, Raini Dutta, Vikas Vats, Rishein Gupta, Rajneesh Jha, Hem Chandra Jha, Pragya Srivastava, Apurb Rashmi Bhengraj, Aruna Singh Mittal

**Affiliations:** Institute of Pathology (ICMR), Safdarjung Hospital Campus, New Delhi 110029, India

## Abstract

*Chlamydia trachomatis* is a leading cause of sexually transmitted infection worldwide and responsible for myriad of immunopathological changes associated with reproductive health. Delayed secretion of proinflammatory chemokine interleukin (IL)-8 is a hallmark of chlamydial infection and is dependent on chlamydial growth. We examined the effect of iron chelators on IL-8 production in HeLa 229 (cervix epitheloid cell, CCL2) cells infected with *C. trachomatis*. IL-8 production was induced by Iron chelator DFO and Mimosine, however, synergy with chlamydial infection was obtained with DFO only. Temporal expression of proinflammatory secreted cytokines IL-1beta, TNF-alpha, and IL-8 did not show synchrony in *Chlamydia trachomatis* infected cells. Secretion of IL-8 from Hela cells infected with *C. trachomatis* was not dependent on IL-1 beta and TNF- alpha induction. These results indicate towards involvement of iron in chlamydia induced IL-8 production.

## 1. Introduction

*Chlamydia trachomatis* is the most common sexually transmitted pathogen which causes severe sequelae in women including pelvic inflammatory disease, ectopic pregnancy, and infertility [1–4]. *C. trachomatis *is able to infect and multiply within a broad range of eukaryotic cells, including macrophages, smooth muscle, epithelial, and endothelial cells [5]. Chlamydial infection of epithelial cells at mucosal surface produces proinflammatory factors such as Interleukin (IL)-1*α*, IL-6, IL-8, IL-11, GRO-*α*, and granulocyte-macrophage colony-stimulating factor [6–8], which can lead to an acute inflammatory response characterized by neutrophil infiltration to the primary sites of infection, followed by a subepithelial accumulation of mononuclear leukocytes during the chronic phase of infection [9–11]. These cellular responses promote cellular proliferation and tissue damage of affected organs [12]. Most of the invasive bacterial pathogens often induce rapid but transient responses [13]. In contrast *C. trachomatis* infection induces delayed proinflammatory responses especially IL-8 production in epithelial cells and is dependent on bacterial replication [7, 14, 15]. In addition Tumor necrosis factor-alpha (TNF-*α*) and IL-1beta (IL-1*β*) have been reported to be the most powerful inducers of IL-8 in a multitude of cell lines and a major contributor in clearance process for intracellular pathogens [16, 17]. 

Bacterial iron chelator (desferal, deferoxamine mesylate) triggers inflammatory signals, including the production of CXC chemokine IL-8 in human intestinal epithelial cells (IECs) by activating ERK1/2 and p38 kinase pathways [18]. Chlamydia enters into persistence stage in the presence of iron-chelating drug (Desferal), thereby showing its dependence on iron for completion of developmental cycle [19]. Persistence can also be induced by antibiosis and tryptophan starvation induced by penicillin G and IFN-*γ* respectively [20–22]. Earlier model of *C. pneumoniae* persistence showed that after IFN-*γ* and penicillin treatment chlamydia-induced IL-8 expression was inhibited, while it stayed up regulated in iron-depletion [23]. In order to develop appropriate therapeutic regimen, it is essential to define the activation pathways where iron chelation controls IL-8 induction in chlamydial infection.

Thus, this study aimed to reveal (i) involvement of intracellular iron in chlamydia induced production of proinflammatory cytokine IL-8 from HeLa 229 cells and (ii) autocrine effect of TNF-*α* and IL-1*β* in production of IL-8 from *C. trachomatis* infected cells under iron deprived condition.

## 2. Materials and Methods

### 2.1. Materials

All the biochemicals were purchased from Sigma-Aldrich (Saint Louis, USA) unless otherwise mentioned: (i) Deferoxamine mesylate (DFO)—bacterial iron chelator; (ii) Mimosine—a plant based iron chelator; (iii) Ferric ammonium citrate (FAC)—iron source; (iv) Glucose oxidase (GO)—oxidant; (v) DETA-NANOate—nitric oxide producer; (vi) Pyrrolidin dithiocarbamate (PDTC)—nitric oxide inhibitor; (vii) Ebselen—antioxidant.

### 2.2. Propagation of *Chlamydia Trachomatis*

HeLa 229 cervix epitheloid cells (CCL2) were maintained in EMEM containing 10 *μ*g/mL gentamicin and supplemented with 10% fetal bovine serum. Further HeLa cells maintained in 50 cm^2^ culture flask were infected with *C. trachomatis* serovar D for propagation. After 60 hours postinfection infected cells were scraped and sonicated (30 cycles of 0.6/0.4 seconds) to release elementary bodies (EBs) followed by centrifugation at 3000Xg to remove cellular debris. Supernatant containing EBs was purified on discontinuous gradients of Renograffin (Squibb, Montreal, Canada) as described previously [24]. Purified elementary bodies (EBs) were resuspended in isotonic sucrose-phosphate-glutamate (SPG) buffer and stored at −80°C. The infectivity of purified EBs was titrated by counting chlamydial inclusion forming units (IFUs) on the monolayer of HeLa 229 cells grown in a 96-well plate.

### 2.3. Infection

Subconfluent monolayer of HeLa cells in 12 well plates was inoculated with EBs at multiplicities of infection (moi) 2. The plates were then rocked for 2 hours at 37°C for homogenous infection after which the extracellular bacteria were removed by washing with phosphate buffered saline (PBS). Subsequently cells were cultured in Delbecco's Minimum Essential Media (Sigma, Saint Louis, USA) containing 10% Fetal bovine Serum (PAA Laboratories GmbH, Pasching, Austria), 10 *μ*g/mL gentamicin (Sigma, Saint Louis, USA), 1 *μ*g/mL Amphotericin B (Sigma, Saint Louis, USA) and incubated at 37°C in 5% CO_2_ environment. After 2 hours of infection, cells were treated with 0.5 mM deferoxamine (DFO), 1 mM mimosine, and 0.5 mM ferric ammonium citrate (FAC) at final concentration in different wells with their respective control. Further DETA-NANOate (1 mM) and glucose oxidase (10 U/mL) was added in combination with DFO, Mimosine and FAC. Antioxidant ebselen (2.5 mM) and PDTC (1 mM) added respective wells along with their controls and analysis was performed with 48 hpi supernatant. Supernatant of mock and CT infected cells were collected at 6, 12, 24, 30, 48, 72, and 96 hpi, centrifuged (Rota 4R-B/F, Plastocraft, Mumbai, India) at 12000Xg (10 min, 4°C), and stored at −80°C until cytokine assay.

### 2.4. Cytokine Assays Using ELISA

Levels of secreted cytokines (IL-8, TNF-*α* and IL-1*β*) in culture supernatants were determined using ELISA kit (Pierce Biotechnology Inc, Rockford, USA and (eBiosciences, San Diego, USA)) having detection limit of 2 pg/mL, 4 pg/mL, and 8 pg/mL. All the assays were performed in duplicate according to manufacturer's instructions.

### 2.5. Statistical Analysis

A statistical analysis was performed using GraphPad Prism software (version 5.0). Differences were tested for statistical significance by 2-way repeated measurements analysis of variance (ANOVA) followed by Bonferroni posttest. Every experiment was done twice in triplicate.

## 3. Results

### 3.1. Elevated Level of Exogenous IL-8 in *C. trachomatis* Infected Hela 229 Cells

**Figure 1:**
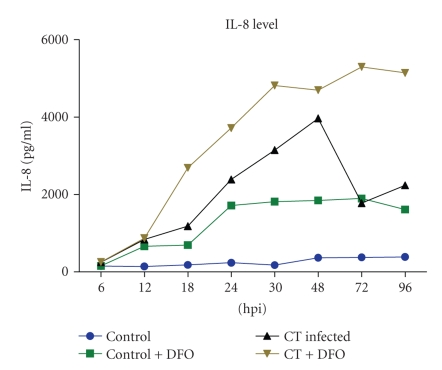
*C. trachomatis* infected HeLa 229 cells showing significantly (*P* < 0.001) increased levels of IL-8 as detected by ELISA in comparison to mock infected cells.

Level of IL-8 was significantly increased (*P* < 0.001) in culture supernatant of *C. trachomatis* infected cells compared to mock infected cells. Further significant increase in IL-8 level was observed in *C. trachomatis* infected cells in addition to iron chelator deferoxamine (DFO). In time dependent study significantly elevated level of IL-8 secretion was detected at 12 hpi, (*P* < 0.001) in *C. trachomatis* infected cells compared to control (mock infected Hela cells). Further in control cells two peaks were seen at 24 and 72 hpi through at 30 hpi representing a biphasic curve, whereas in *C. trachomatis* infected cells secreted IL-8 level represented a monophasic curve which showed consistently significantly increased levels from 12 hpi till 72 hpi (*P* < 0.001) (Figure 1).

### 3.2. Secretion of IL-8 is Independent of Exogenous IL-1*β* and TNF-*α* in Chlamydia Infected Cells

**Figure 2:**
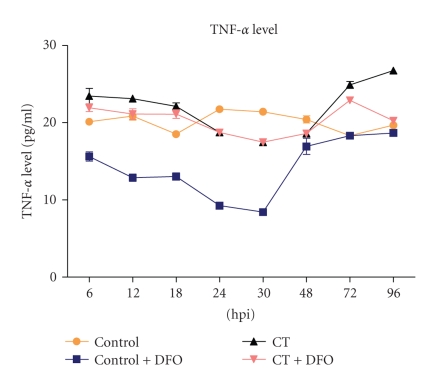
TNF-*α* concentration as determined by ELISA in *C. trachomatis* infected culture supernatant was significantly (*P* < 0.001) increased at early time points (6, 12, and 18 hpi) and late time points (72 and 96 hpi), whereas there was decrease at midtime points (24, 30 and 48 hpi) in comparison to mock infected.

**Figure 3:**
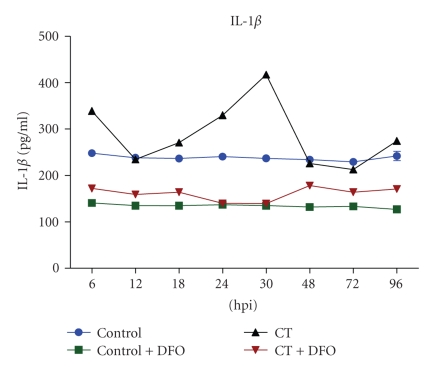
Levels of IL-1*β* in supernatants of *C. trachomatis* infected culture were significantly decreased (*P* < 0.005) at early (6, 12, and 18 hpi) and late (72 and 96 hpi) time points in comparison to mock infected culture, whereas at midtime point no significant (*P* < 0.001) change was observed.

Temporal expression of IL-1*β* and TNF-*α* was assessed to ascertain the involvement of these cytokines in IL-8 induction. There was significant increase (*P* < 0.001) in level of secreted TNF*α* in culture supernatants during early (6, 12, and 18 hpi) and late phases (72 and 96 hpi) (Figure 2) whereas IL-1*β* levels were elevated at 30 hpi (Figure 3). However, at the midphase of *C. trachomatis* growth, level of secreted TNF-*α* declined, whereas IL-1*β* level did not show any deviation from basal level (Figures 2 and 3) and secretions of TNF-*α*, IL-1*β*, and IL-8 did not show synergy at different time points.

### 3.3. IL-8 Expression with Iron Chelator DFO and Mimosine in *C. trachomatis* Infected Hela Cells

**Figure 4:**
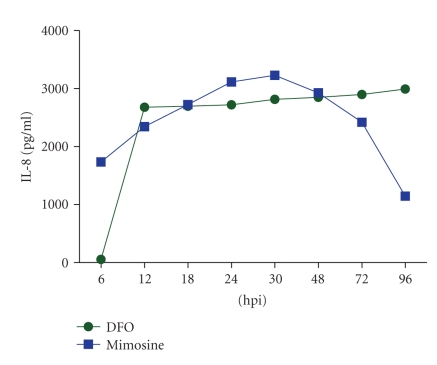
Gradual increase was detected in levels of IL-8 in culture supernatants, highest at 30 hpi; thereafter, subsequent decrease was observed from 48 hpi onwords in *C. trachomatis* infected cells treated with Mimosine. DFO treated cells showed delayed secretion (12 hpi) but consistently elevated levels were detected at all the time point taken.

Mimosine, a plant based iron chelator, showed earlier induction of IL-8 at 6 hpi in *C. trachomatis* infected HeLa cells whereas in presence of DFO there was delayed (12 hpi) induction and was consistent at all time intervals (Figure 4). Gradual increase in IL-8 expression was detected, being highest at 30 hpi which subsequently declined at 48 hpi (Figure 4).

### 3.4. Chlamydia Induced IL-8 Production is Mediated by Reactive Nitrogen Species in Iron Restricted Condition (Evaluated at 48 hpi)

**Figure 5:**
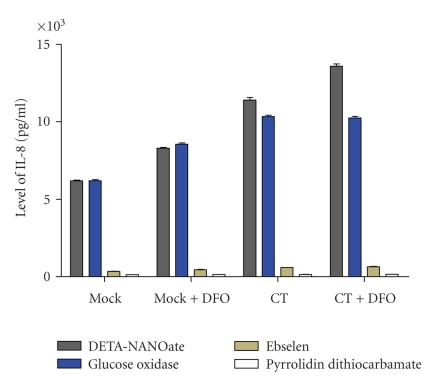
IL-8 induction was significantly (*P* < 0.001) increased in *C. trachomatis* infected HeLa cells after stimulation with DETA-NANOate and there was synergic effect with DFO. Glucose oxidase treatment induced IL-8 production at same degree in both the *C. trachomatis* infected and Mock conditions (*P* < 0.005). Higher inhibition (*P* < 0.05) of IL-8 induction was observed after addition of nitric oxide specific inhibitor PDTC than ebselen.

IL-8 production was significantly increased (*P* < 0.001) in *C. trachomatis* infected HeLa cells after stimulation with DETA-NANOate (Figure 5). Further significant increase (*P* < 0.001, Figure 5) was observed in IL-8 production in mock and CT infected cells upon co-stimulation with DFO and NO. However additive effect of DFO and NANOate was significantly higher (*P* < 0.005) in CT infected cells (Figure 5). Moreover glucose oxidase induced IL-8 production irrespective of CT infected or Mock (*P* < 0.005, Figure 5). Ebselen and PDTC decreased the production of IL-8 in all the conditions and significantly higher inhibition (*P* < 0.05, Figure 5) was observed with PDTC (Figure 5).

## 4. Discussion

Chlamydial infections such as sexually transmitted or respiratory diseases occur frequently; however, course of these illnesses tends to be mild. Hence, they often pass unrecognized and untreated. These predominantly mild acute infections are considered as possible starting points of other chronic diseases, such as tubal inflammation with infertility, or reactive arthritis for *C. trachomatis* [25–27]. Disease outcome of Chlamydial infection is predominantly governed by mediators of inflammation and IL-8 leading the belligerence. Elevated level of IL-8 is dependent on chlamydial growth [7] and it is reported that in case of deferoxamine induced persistence, level of IL-8 remains higher [23]. These observations raise a question if chlamydia is directly involved in IL-8 induction or else intracellular level of iron is also a contributor?

 In our study it was observed that in *C. trachomatis* infected HeLa 229 cells, level of secretory IL-8 increased significantly (*P* < 0.001) at 12 hpi till 72 hpi in comparison to mock infected cells showing thereby the probable involvement of early phase of chlamydial differentiation and metabolic activities responsible for induction and secretion of IL-8. This is in accordance with earlier study [28] wherein it has been shown that *C. trachomatis* and *C. psittaci* up regulate mRNA expression and secretion of the proinflammatory cytokines like IL-8, GRO*α*, GM-CSF, and IL-6. Further IL-8 production in HeLa 229 cells infected with *C. trachomatis* was significantly delayed (from 24 hpi), unlike most invasive microorganisms that promote rapid but transient inflammatory response [7]. Elevated level of IL-8 might help epithelial cells at the mucosal surface to communicate with professional immune cells in response to pathogenic insult.

Further it was observed that IL-8 induction is independent of TNF-*α* and IL-1*β* in *C. trachomatis* infected HeLa 229 cells. Earlier studies had also suggested that TNF-*α* and IL-1*β* act as costimulatory signal for IL-8 induction in NF-*κ*B dependent pathway [29]. Chlamydiae contains a tail serine protease (Tsp) that selectively cleaves the p65/RelA subunit of NF-*κ*B to potentially interfere with host inflammatory response [30]. In a recent study lower level of TNF receptor 1 was observed on cell surface of chlamydia infected cells, inhibiting TNF mediated signaling [31] thereby enticing the possibility of TNF-*α* and IL1*β* independent expression of IL-8 in *C. trachomatis* infected HeLa 229 cells. 

 We also demonstrated that chlamydial infection acts synergistically with DFO to activate IL-8 secretion. Intracellular level of available iron has been shown to modulate inflammatory mediators and to regulate inflammatory process in several cell types. Two important signaling pathways represented by p38 and ERK1/2 are required for DFO-induced IL-8 secretion [18, 32–34]. IL-8 induced by *C. trachomatis* has been shown to be dependent on ERK and independent of p38 and Jun N-terminal MAPK [35] showing thereby that chlamydial infection and DFO follow the same course for IL-8 activation. However, further study of posttranscriptional mechanisms for DFO induced IL-8 involving p38 or ERK is warranted.

In our study DFO-induced IL-8 was increased by DETA-NANOate and H_2_O_2_ although extent of stimulation is less than that of NO in HeLa cells infected with *C. trachomatis*. It has been reported that NO is the initial factor in the signaling cascade that mediates IL-8 gene transcriptional activation by DFO. NF-*κ*B is also an important transcriptional factor for IL-8 production in cancer cells. PDTC, one of the most effective inhibitors of NF-*κ*B, inhibits the NO-dependent induction of the IL-8 gene [36]. It may be elucidating that NO plays important role in IL-8 induction in *C. trachomatis* infected cells treated with DFO. 

In *C. trachomatis* infected cells, Mimosine treatment showed gradual increase and subsequent decrease in IL-8 expression. Conversely, delayed induction and consistent expression of IL-8 expression were observed with DFO. Thus synergy was observed only with DFO having bacterial origin, indicating that level of intracellular iron plays an important role in induction of proinflammatory responses. Further it may indicate towards involvement of iron sequestration mechanism of chlamydial origin and acquisition of iron from labile iron pool. Results of this study indicate the involvement of intracellular iron in activation of IL-8 in chlamydia infected HeLa 229 cells.
